# Act now! Critical care roles and obligations during an urban war

**DOI:** 10.1186/s13054-022-03951-z

**Published:** 2022-03-21

**Authors:** Kateryna Bielka, Katarzyna Kotfis, Ronald Poropatich, Michael R. Pinsky

**Affiliations:** 1 Anesthesiology and Intensive Care Department, Bogomolets National Medical University, Kyiv, Ukraine; 2 Department of Anesthesiology, Intensive Therapy and Acute Intoxications, Pomeranian Medical University, Szczecin, Poland; 3 Department of Medicine, Center for Military Medicine Research, University of Pittsburgh, Pittsburgh, PA USA; 4 Department of Critical Care Medicine, Center for Military Medicine Research, University of Pittsburgh, Pittsburgh, PA USA

People trapped in an urban war zone are at increased risk of not only physical and mental trauma but lack of fundamental healthcare resources to treat all other diseases they may have or develop. These risks are often made worse by the war-associated fundamental loss of infrastructure, including access to freshwater, food and routine medical resources. If war is protracted beyond a few days, aggressors often specifically target these civilian systems to break the will of the population. Following the recent invasion of Ukraine by Russian army forces, resistance by Ukrainian forces prevented a rapid victory. Not surprisingly, this led to a change in Russian military strategy to attack unarmed civilian centers and the infrastructure of its major cities. The resultant human cost to the Ukraine people is unmeasured but must be extremely large. Massive numbers of Ukrainian refugees, mainly women and children, are crossing the border into Poland, Slovakia, Hungary, Romania, and Moldova and further on to other countries to seek refuge from the war.

We, the non-combatant Western communities, have an obligation to immediately help these refugees and those who are unable to escape the ravages of urban war. What can and should be done is a matter of debate, but prior experience with the COVID-19 pandemic, disaster medicine and telemedicine suggest ways such help can be delivered quickly, efficiently and effectively. Disasters, whether natural or manmade, are unpredictable and require a coordinated and experienced expert team. There is a need for an integrated and structured approach for all three well-defined phases of disaster management (pre-, during and post-disaster) that must be addressed to ameliorate the impact on life and the necessary steps for recovery. However, once started, the medical support integration needs to be done rapidly, as summarized in Table 1 and described below.

**Table 1:** Population, support and institutions/site for acute healthcare relief during an urban war

Process	Population	Support	Institutions/sites
Refugees retrieval	Refugees from war area, mainly women and children	Shelter, clothing, freshwater, food and warmth Restoring normality by enrolling in schools and providing jobs to minimize post-traumatic stress	International relief agencies International humanitarian cooperation
Immediate medical care outside war zones	War casualties retrieved from war zones Refugees leaving a war zone in need of immediate medical support	Field intensive care units (playing fields and unoccupied warehouses) staffed by less skilled aids overseen by some trained physicians, nurses, respiratory therapist and allied healthcare providers. Run by military units during the initial stages of triage for major natural disasters Temporary hospitals, affiliated with permanent hospitals, already equipped with all the necessary infrastructure, and employing skilled professionals, that can be used for patients requiring immediate life support Transfer to permanent healthcare structures for patients in need of prolonged care	Neutral neighborhood countries (i.e., eastern border of Poland)
Distributive universal care	Refugees leaving a war zone unable to be transferred Casualties who are unable or unwilling to leave their homes and country	Smaller telemedicine-based treatment stations across the affected regions linked to remote central expert clinical decision support Rapid deployment of expert care triage across a wide region of land where no other services are presently available Multinational Telemedicine System Experts (MnTS) by establishing the network and a concept of operations, to be used in disaster management between countries	Multinational Telemedicine System (MnTS) for disaster response from the North Atlantic Treaty Organization (NATO), under the auspices of the Science for Peace and Security Program, in Lviv, Ukraine

First, based on disaster medicine principles, we know that at the minimum these refugees need clothing, shelter, freshwater, food and warmth. For the children, to the extent possible attempts at normality by enrolling in schools minimized post-traumatic stress. Many international relief agencies in all neighborhood countries, like the International Red Cross, support these issues.

But what of the healthcare issues of a massive population disrupted and displaced? From the experiences with the COVID-19 pandemic all around the world, where all healthcare services were rapidly overwhelmed, we learned that it is possible to set up field intensive care units in playing fields and unoccupied warehouses, staffed by less skilled aids overseen by some trained physicians, nurses, respiratory therapist and allied healthcare providers. From a logistical perspective, it makes sense to place such systems far forward but in neutral countries, like on the eastern border of Poland. Such field hospitals to treat civilians are often run by military units during the initial stages of triage for major natural disasters. The COVID-19 pandemic has provided us with temporary hospitals, affiliated with permanent hospitals, already equipped with all the necessary infrastructure, and employing skilled professionals, that can be used for needs of patients requiring immediate life support. These systems would then transfer to permanent healthcare structures those patients in need of prolonged care. But these field hospitals cannot support the medical needs of a massive wave of refugees leaving a war zone, nor support those who are unable or unwilling to leave their homes and country.

For this larger distributive universal care need, other approaches are needed. Creating smaller telemedicine-based treatment stations across the affected regions linked to remote central expert clinical decision support represents a realistic solution for rapid deployment of expert care triage across a wide region of land where no other services are presently available. Since the 1990s, telemedicine has been integrated in some form of disaster response. This adoption and integration has been shown to be effective [1–3]. In 2015, the North Atlantic Treaty Organization (NATO), under the auspices of the Science for Peace and Security Program, developed and deployed to Lviv, Ukraine, a Multinational Telemedicine System (MnTS) for disaster response [4]. A group of subject matter experts from Europe and the USA developed the MnTS by establishing the network and a concept of operations, to be used in disaster management between countries.

The MnTS is an integrated system that includes personnel, hardware, communication protocols, portable power generation, medical kits, and Web-based tools. In 2015, the MnTS was successfully tested in the Euro-Atlantic Disaster Response Coordination Centre’s Exercises in Ukraine. The field exercise tested and validated the MnTS and identified areas of improvement. For the current Ukraine conflict, major infrastructure damage to the power grid and limited medical resources and logistics will hamper tele-consultation support from the international community. The NATO experience gained from this exercise and the current NATO Telemedicine Expert Panel are well positioned to organize a consolidated MnTS that other well-intentioned civilian health providers could join to augment the clinical response and expertise.

The medical needs for the Ukraine people will only increase over time, and we must act now to organize and generate a committed team of international military–civilian providers that support both Ukraine refugees in border countries and in-country casualties. Immediate, coordinated response between our governments, international aid societies and international critical care medical societies need to unify in this effort to minimize this existing man-made humanitarian disaster. Otherwise, we will reap greater long-term effects of death, disease and emotional dysfunction that urban warfare always brings.

## Data Availability

N/A.
